# Caregiver-reported sleep problems and suicidality in autistic youth: findings from a sleep diary study

**DOI:** 10.3389/fpsyt.2026.1731938

**Published:** 2026-05-04

**Authors:** Hangsel Sanguino, Chris A. Clark, Kailyn M. Turner, Stephanie J. Howe, Carly A. McMorris

**Affiliations:** 1 Werklund School of Education, University of Calgary, Calgary, AB, Canada; 2 Alberta Children’s Hospital Research Institute (ACHRI), Calgary, AB, Canada

**Keywords:** autism, sleep problems, suicidality, young adult, youth

## Abstract

**Background:**

Sleep problems, including daytime sleepiness, short sleep duration, and poor sleep quality, are strongly associated with suicidality in non-autistic people. Sleep difficulties are also common in Autistic children and adolescents (herein, youth), affecting 40-80%, and may contribute to their elevated suicide risk, which emerges as early as 10 years of age and is 3.5 times higher than the general population. Despite the strong link between sleep difficulties and suicidality in non-autistic people, no study has examined this association in Autistic youth. This cross-sectional online survey addressed this gap by investigating links between sleep and suicidality in Autistic youth using caregiver reports and daily sleep diaries.

**Methods:**

Fifty-one caregivers (87.7% female, *M*_age_ = 40.78, *SD* = 8.04) of Autistic youth aged 7–26 years (*M*_age_ = 12.02, *SD* = 4.14) completed an online survey including the Children’s Sleep Habits Questionnaire and a modified Columbia-Suicide Severity Rating Scale (C-SSRS). Caregivers also completed sleep diaries about their child’s sleep each morning upon awakening for 7 days.

**Results:**

Most caregivers (88%) reported their Autistic youth had sleep problems, and 64.7% endorsed at least some suicidal behaviour. Daytime sleepiness (*B* = 0.19, *SE* = 0.08, *p* = .011) and sleep quality (*B* = -1.19, *SE* = 0.585, *p* = .04) were related to suicidal ideation, such that caregivers of Autistic youth with poorer sleep were more likely to report that their youth experienced suicidal ideation. Daytime sleepiness was related to both lifetime (*B* = 0.832, *SE* = 0.238, *p* <.001) and recent C-SSRS total scores (*B* = 0.691, *SE* = 0.240, *p* <.01), while, unexpectedly, total sleep problems were not associated with total suicidality scores.

**Conclusion:**

Specific sleep difficulties, particularly daytime sleepiness and sleep quality, may uniquely contribute to caregiver-reported suicidality in Autistic youth. With few effective suicide interventions for Autistic individuals, these findings provide critical insight into the sleep-suicide connection that can help inform targeted prevention strategies.

## 1 Introduction

Suicidal thoughts and behaviours (“suicidality” hereafter) are common and have been recognised as a major concern in Autistic individuals (1–3). It is estimated that both ideation and attempts occur in approximately 66% of Autistic adults (1). Among Autistic children and adolescents (herein, “youth”), one in four reports experiencing suicidal ideation, and almost one in ten attempt suicide, a rate almost four times higher than that of their non-autistic peers (2–4). Identifying risk and protective factors for suicidality in Autistic youth is critical for creating effective prevention and intervention strategies. In this article, we use identity-first language (i.e. ‘Autistic youth’) to reflect the language preferences expressed by many members of the autism community.

Various social and psychological factors may heighten the risk of suicide in Autistic adults, including alexithymia (5), executive functioning difficulties (6, 7), loneliness and lack of social support (8), reduced feelings of belonging (9), and social masking (10). In Autistic youth, some demographic factors such as ethnicity, age, and gender have been linked to suicidal behaviour (11–13). For instance, Conner and colleagues (11) found that Hispanic/Latine Autistic youth are more likely to have suicidal behaviours and past attempts as reported by their caregivers. Older adolescents and females appear to be at increased risk of suicide in Autistic populations (12–14).

Similarly, So et al. (13) reported that risk of suicide or self-harm was the most common reason for psychiatric emergency referral among Autistic youth (60.9% of girls; 48.0% of boys). In their study, suicide risk and self-harm were recorded as a single referral category and were not differentiated as suicidal versus non-suicidal self-harm. It is possible that underlying symptoms or transdiagnostic features, such as emotion regulation, altered stress responses, and most importantly for the present study, sleep difficulties, may contribute to suicidality in Autistic youth (4, 15, 16). Sleep problems are particularly relevant in this context, as they are exceedingly common in Autistic youth and adults (17–19) and represent a modifiable risk factor (20), making them a potential target for suicide prevention efforts.

Sleep problems are increasingly recognised as a common and co-occurring symptom in Autistic youth, with 50-80% of youth experiencing sleep problems, including falling asleep, staying asleep, early morning wakings, daytime sleepiness, and night terrors (10). Most Autistic youth experience delayed sleep onset, night wakings, shorter sleep durations, disrupted circadian rhythms, poor quality of sleep, and early morning awakenings (14). Bedtime resistance, characterised as behavioural difficulties around settling to bed alone, is also quite common in Autistic youth (16, 18–20), and could be due to greater sleep anxiety, hyperactivity, and general dysregulation often experienced by Autistic children. In their recent systematic review, Carmassi et al. (21) propose a theoretical framework, including genetic and biological factors, that might help to explain the bidirectional connection between sleep problems and autism. For example, genetic studies demonstrate that Autistic individuals might have polymorphisms and mutations in circadian clock genes, leading to irregular or delayed sleep phases, as well as atypicalities in genes related to melatonin production (21). These authors further suggest that mutations in circadian clock genes contribute to sleep challenges, which in turn could exacerbate autistic traits (21).

Sleep problems are a significant risk factor for suicidality in non-autistic people, with several meta-analyses showing this association, and effect sizes ranging from small to medium (14, 22, 23). In their systematic review, Bernert and colleagues (14) identified that sleep problems, such as nightmares and insomnia, characterised by difficulties initiating and maintaining sleep, were associated with greater suicidal risk in both non-clinical and psychiatric patient populations. Similarly, Liu and colleagues (23) reported that adolescents with sleep problems were at a 79% higher risk of suicidal ideation than youth with no sleep problems. Furthermore, these authors found that more frequent nightmares increased the risk of suicidal thoughts by approximately 1.5 times, and an increase in attempts by 4 times. This growing body of evidence highlights that insomnia is highly prevalent in people experiencing suicide ideation, even after controlling for depressive symptoms (24). Further, in the general population, other sleep domains, such as daytime sleepiness, short sleep duration, and poor sleep quality, are associated with all aspects of suicidality (i.e., suicidal ideation, attempts, and death by suicide) (22–24). Shorter sleep (less than 8 hours) significantly increases the risk of suicide attempts, even after adjusting for gender, age, and depression (25). Lastly, longer sleep duration is associated with a higher likelihood of recent (past week) suicidal ideation in non-autistic people. Together, these findings highlight that a wide range of sleep difficulties may play a role in the development of suicidality.

To date, few studies have examined the association between sleep and suicidality in Autistic youth. Mayes and colleagues (3) found significant differences in the frequency of caregiver-reported ideation and attempts in Autistic children with and without sleep problems. In their cohort of almost 800 Autistic children, 364 older children (i.e., age > 10 years) with sleep problems were reported to have significantly higher ideation and attempts (18.6%) compared to a group with no sleep problems (*n* = 435, 9.7%). Similarly, Hochard and colleagues (25) reported that shorter sleep duration, alongside elevated autistic traits, was associated with greater suicidality. Existing literature has also been limited methodologically, with research to date only utilising single-item tools to identify ideation or attempts. By doing so, existing research has failed to use a comprehensive score to capture the full spectrum of suicidality, including severity and intensity, limiting our understanding of what specific aspects of sleep disturbances contribute to the presence, severity, frequency, and intensity of suicidality, information that is critical for the development of appropriate suicide prevention approaches.

To expand existing literature, this study examined the relation between caregiver-reported sleep problems and suicidality in Autistic youth, while controlling for age and sex, both established risk factors for suicide, and employing a multi-method approach to assessing sleep. Additionally, the study investigated whether seven-day sleep patterns using sleep diaries differed between participants with and without suicidality. Building on previous research in non-autistic youth (14, 23), it was hypothesised that sleep problems in Autistic youth would be linked to greater suicidality, with those experiencing or having a history of suicidality showing shorter sleep duration, more night awakenings, poorer sleep quality, and later sleep times over the seven days.

## 2 Materials and methods

### 2.1 Setting

This project was approved by the Conjoint Health Research Ethics Board (CHREB) at the University of Calgary. Caregivers were recruited from clinics and autism-specific community agencies in Calgary, between January 2020 and May 2021.

### 2.2 Participants

This study consisted of 57 caregivers (parents and legal guardians) of Autistic youth enrolled in a larger study about characterising suicidal thoughts and behaviours among Autistic youth, with some of the findings being presented elsewhere (26). Inclusion criteria included being the caregiver of an Autistic youth or young adult between the ages of 7 and 26 years of age and fluency in English. The broad developmental age range was included to capture the variability in sleep and suicidality that may emerge across this developmental period. The average age of caregivers was 40.74 years (*SD* = 8.041), while the average age of the Autistic youth was 12.02 years (*SD* = 4.14). Most caregivers were married mothers (*n* = 49, 86%) with a mean household income range of $100,000 and over CAD/year. Two participants did not report household income. Most participants identified as White (87.7%). Most caregivers reported that Autistic youth had a co-occurring diagnosis (77.2%), with the most common being attention-deficit/hyperactivity disorder (ADHD; 36.8%) and anxiety disorders (generalised anxiety disorder, panic disorder/agoraphobia, social anxiety disorder, and others; 21.1%). Participant demographic information is reported in **Table 1**.

**Table 1:** Demographic characteristics (n=57).

Variable	Mean (SD)	n (%)
Child Demographics		
Age	12.1 (4.17)	
Gender		
Female		15 (26.3)
Male		42 (73.7)
Ethnicity		
White or Caucasian		50 (87.7)
South Asian (East Indian, Pakistani, Sri Lankan, etc.)		3 (5.3)
Asian		1 (1.8)
Other (e.g., Black, Somali, Arab, and Iranian)		3 (5.2)
Age of diagnosis	6.42 (3.27)	
Co-occurring mental health diagnoses		
ADHD		21 (36.8)
Anxiety disorders		12 (21.1)
Depression		2 (3.5)
Other		9 (15.8)
No formal diagnosis		13 (22.8)
Sleep Medication		
Yes		25 (43.9)
No		32 (56.1)
Caregiver Demographics		
Age (*n*=56)	41.27 (6.71)	
Education Level		
High school diploma or equivalent		6 (10.5)
Diploma or certificate below bachelor level		26 (45.6)
Bachelor’s Degree or higher		25 (43.9)
Total Annual Household Income (n = 55) ^b^		
less than $49,000		8 (15.4)
$49,000 - $79,000		18 (32.7)
over $80,000		29 (52.7)

Values are reported as Mean (*SD*) or *n* (%). *n*, sample size; SD, standard deviation; ADHD, attention-deficit/hyperactivity disorder.

### 2.3 Measures

*The Children’s Sleep Habits Questionnaire* (27). The CSHQ was used during the baseline assessment to assess youth’s sleep problems. It is a 33-item caregiver-reported questionnaire for school-aged children ages 4 to 10 years. However, it has been widely used in research with youth ranging from 2 to 18 years of age and has been found to accurately capture sleep difficulties in Autistic youth (28). Further, the CSHQ has been applied and shown to accurately detect sleep difficulties in adolescents aged 11 to 18 years (20, 27–31). The CSHQ provides a total score and eight subscales: Bedtime resistance (6 items), Sleep-onset delay (1 item), Sleep duration (3 items), Sleep anxiety (4 items), Night wakening (3 items), Parasomnias (7 items), Sleep disordered breathing (3 items), and Daytime sleepiness (8 items). All items are answered on a 3-point Likert scale from “rarely” (i.e., never or 1 time during the week) to “usually” (i.e., 5 or more times in a week), with higher scores indicating more sleep problems. One additional question gathered information about the average length of awakenings during the last week. Duration of night awakenings was manually scored (i.e., 1 = no, 2 = yes) for wakings lasting more than five minutes (29). A total score of > 41 is the cut-off to indicate children at risk for paediatric sleep disorders and has been shown to accurately identify 80% of children with a clinically diagnosed sleep disorder (27, 30). The CSHQ has been documented to have good internal consistency, with a Cronbach’s α of.81 (27, 29). In our study, the CSHQ Cronbach’s α values were: (Bedtime resistance (*α* = .78), Sleep duration (α = .80), Sleep anxiety (α = .76), Night wakening (α = .60), Parasomnias (α = .60), Sleep disordered breathing (α = .88), Daytime sleepiness (α = .83)). For the current study’s analysis, both the total score and the subscale scores on the CSHQ were used, with higher scores indicating more severe sleep problems in each domain. This approach allowed for a comprehensive assessment of sleep difficulties across multiple sleep domains.

***Sleep Diary.*** Online daily sleep diaries were completed by caregivers each morning upon awakening for a one-week (seven-day) period using the platform REDCap, which distributed email links to the surveys. Caregivers answered a series of questions about their youth’s bedtime, wake-up time, assumed total sleep time (TST; in hours), napping (number of naps), and whether they seemed refreshed or fatigued the following morning (e.g., 3 = “my child woke up refreshed”, 1 = “my child woke up fatigued”). Sleep diaries are shown to provide reliable and valid estimates of subjective sleep parameters (29). For the present paper’s analyses, the data from the seven days of surveys were combined into the mean and standard deviation of three measures across the entire period: TST, sleep onset time and sleep quality as measured by the “refreshness” ratings. Earlier sleep onset times (i.e., lower recorded values) and higher sleep quality were both representative of better sleep. TST was more complex, as there are recommended amounts of sleep based on age and having either too much or insufficient sleep is typically representative of sleep issues (32, 33).

*Columbia-Suicide Severity Rating Scale (C-SSRS)* (34). A modified version of the C-SSRS was used to measure suicidality. The C-SSRS was selected because it is a comprehensive tool for assessing multiple dimensions of suicidality (e.g., ideation severity, intensity, behaviour, and lethality) in youth (34, 35). The C-SSRS is typically administered as a semi-structured interview by a trained interviewer. However, in the current study, the C-SSRS was adapted for caregiver report, with item wording changed so that caregivers reported on their child’s suicidality rather than the youth completing the interview. For example, the item “Have you wished you were dead or wished you could go to sleep and never wake up?” was modified to “Has your child wished they were dead or wished they could go to sleep and never wake up?” No items were added or removed. Caregiver-report of suicidality was selected because data collection for this study occurred during the COVID-19 pandemic and required a pivot to fully remote data collection. Caregiver-report also mitigates potential comprehension or reporting issues due to young age or autistic traits (e.g., alexithymia, difficulty with abstract future thinking) that may compromise the validity of existing self-report tools (35).

This approach is consistent with prior research that has used caregiver-reported versions of the C-SSRS in web-based formats (36). Flamarique and colleagues compared a web-based caregiver version of the C-SSRS to the standard C-SSRS interview using Receiver Operating Characteristic (ROC) analysis (36). The results showed that the web-based caregiver version had fair accuracy (ROC area under the curve 0.76). Additionally, the correlation between the web-based caregiver version and the standard C-SSRS was moderately strong (*r* = 0.548, *p* < 0.001) (36). Given that the C-SSRS was originally developed as an assessment tool for suicidal ideation and behaviour, no instructions for a total score have been developed.

The self-report interview contains a series of questions related to suicidal ideation, intensity of suicidal ideation, and suicidal behaviour at a time when the person felt most suicidal (lifetime) and within the past one month (recent). Five yes/no questions inquire about whether the individual: has wished to be dead; has thoughts about dying without a specific method or plan (i.e., non-specific active suicidal thoughts); has thoughts about dying with a specific method, but no plan (i.e., active suicidal ideation with methods without intent to act); has thoughts about dying with some intention to act (i.e., active suicidal ideation with some intent to act without specific plan); and has thoughts about dying with the details of a specific method and intention to act (i.e., active suicidal ideation with specific plan and intent) (34). Five constructs related to intensity of suicidal ideation were explored: frequency (1 = <once a week to 5 = many times a day); duration (1 = a few seconds/minutes to 5 = more than 8 hours); controllability (1 = easily able to control to 5 = unable to control); deterrents (1 = deterrents definitely stopped from attempting suicide to 5 = deterrents definitely did not stop from attempting suicide); and reasons for ideation (1 = completely to get attention, revenge or a reaction from others to 5 = completely to end pain).

A series of yes/no questions also explores suicidal behaviour in the C-SSRS, where suicide attempts (i.e., the person engaged in a self-injurious act committed with a wish to die as a result of the act) are distinguished from interrupted or aborted attempts. Interrupted suicide attempts include events where the person is stopped by an outside circumstance (e.g., another person) from engaging in a self-injurious act that otherwise would have been committed with the intention to die. Aborted or self-interrupted attempts include instances where the person took steps towards making a suicide attempt but stopped themselves before starting the self-injurious act. Additionally, a yes/no question inquires about non-suicidal self-injurious behaviour (NSSI), where the individual may cause themselves harm without the intention to die. Lastly, the C-SSRS suicidal behaviour section includes a yes/no question about preparatory acts taken towards making a suicide attempt, such as assembling a specific method (e.g., buying pills, purchasing a gun) or preparing for one’s death by suicide (e.g., giving things away, writing a suicide note). Lethality or medical damage caused by a suicide attempt is also rated from 0 (no physical or very minor physical damage) to 5 (death).

For this study, we used recommendations from Lindh to create a total suicidality score of 0–42 using responses to items from all subcategories of the measure (37). This total score was used as an indication of overall suicidality, including the presence and intensity of suicidal ideation, the overall severity of suicidal ideation, suicidal behaviours, including past attempts or preparation, and the severity of past attempts in the present study. Although this scoring framework was originally developed in psychiatric samples, the multidimensional structure of the C-SSRS allows for a comprehensive approach to quantifying suicidality (35).

For each subcategory of suicidality, a separate subscale score was calculated before summing the subscales to create a total score. For the severity of suicidal ideation, the maximum (i.e., most severe) ideation present was the suicidal ideation score. For suicidal behaviour, the number of recorded attempts was used to score the subscale: 0 (no attempts), 1 (1–2 attempts) and 2 (three or more attempts). If participants engaged in NSSI or preparatory acts, they also received 1 additional point each on the behaviour’s subscale. A continuous score for both the intensity of suicidal ideation and the lethality of suicidal behaviour (including both actual and potential lethality) was created following C-SSRS guidelines. This approach aligns with prior work by Lindh et al., who used continuous scoring of C-SSRS ideation intensity and total scores to predict short-term suicidal outcomes in a psychiatric population (37, 38).

### 2.4 Procedure

All online questionnaires were completed by caregivers using the REDCap survey platform. Caregivers reported basic demographic information, including their age and background, as well as the youth’s age, ethnicity, autism diagnosis (year, healthcare provider), service utilisation, and hospital visits. Caregivers were also asked to provide information about their youth’s mental health diagnoses and a list of current medications. Next, caregivers completed a baseline survey that included the above measures on sleep (CSHQ) and suicide (C-SSRS). The day after completing the baseline measures, caregivers were sent a link to the daily sleep diary. Caregivers were then asked to complete the seven-day sleep diary for their youth’s sleep. Surveys were sent to participants using the REDCap platform, which automatically generated and emailed unique links to the participants every morning for a seven-day period.

### 2.5 Data analysis

Statistical analyses were performed using the open-source statistical program jamovi (Version 1.6, jamovi Team). After calculating basic demographic and descriptive statistics, the main analysis consisted of two parts. First, a series of binomial logistic regressions were calculated to determine if any measure of sleep was a significant predictor of group membership (experienced suicidality or not) on multiple dichotomous variables on the C-SSRS assessing suicidal ideation and behaviour. In particular, the dichotomous suicidality variables included the caregiver’s answers to yes or no questions on the C-SSRS assessing both lifetime and recent suicidal ideation, self-injury, non-suicidal self-harm, and preparation to attempt suicide by the Autistic youth. The sleep variables included the total scores and subscales of the CSHQ collected at baseline, as well as the mean ratings and variability of TST, sleep onset, and sleep quality collected over the seven days of surveys. The reference level for all regressions was that the participant reported a no. Assumptions for regression analyses were examined before conducting the models.

Following this, data from participants who did not endorse suicidality were removed and a second series of analyses were calculated to determine if either baseline sleep (as per the CSHQ total score and subscales scores) or the mean ratings and variability of sleep variables across the seven days predicted the total suicidality score in Autistic youth who had reported a history of suicidal ideation on the C-SSRS represented by the total severity score. Non-suicidal participants were removed from this analysis to reduce floor effects, as they would all have a total score of 0. These analyses were conducted using linear regressions with the dependent variables of lifetime and recent total scores on the C-SSRS. As a sensitivity analysis, additional models were also run, controlling for age as a covariate, which produced the same results as the reported models, and as such were not included.

## 3 Results

### 3.1 Suicidality

Caregiver-reported suicidal thoughts and behaviours are presented in **Table 2**. Of the 57 caregivers who completed the questionnaire, 33 (57.9%) reported that their Autistic youth experienced lifetime suicidal ideation. Six participants (10%) reported that their Autistic youth had attempted suicide in their lifetime, ranging from 1 attempt to 3 attempts, while 26 (45%) reported that their Autistic youth had engaged in non-suicidal self-injury (NSSI). Finally, 7 participants (12%) reported that their Autistic youth had engaged in preparatory behaviour towards a suicide attempt, including gathering materials or writing a suicide note.

**Table 2:** Characteristics of caregiver-reported suicidal thoughts according to C-SSRS items (n=33).

Scale/item	n (%)
Suicidal ideation - *the young person*:	
Has wished to be dead	35 (100%)
Has thought about dying without a specific method or plan	24 (72.7%)
Has thought about dying with a specific method, but no plan (i.e., time, place)	19 (56.6*%*)
Has thought about dying with some intention to act on their thoughts	18 (54.5*%*)
Has thought about dying with details of a specific method and plan fully worked out and intention to act	14 (42.4)
Frequency - *the young person had suicidal thoughts*:	
<once a week	13 (43.3)
Once a week	2 (6.7)
2–5 times a week	7 (23.3)
Daily or almost daily	4 (13.3)
Many times a day	4 (13.3)
Duration - *the young person’s suicidal thoughts lasted*:	
A few minutes/seconds	3 (10.0)
<1 hour	8 (26.7)
1–4 hours	14 (46.7)
4-8 hours	3 (10.0)
>8 hours	2 (6.7)
Controllability - *the young person could stop thinking about wanting to die*:	
Easily	3 (10.0)
With little difficulty	6 (20.0)
With some difficulty	4 (13.3)
With a lot of difficulty	7 (23.3)
Is unable to control their thoughts	6 (20.0)
Doesn’t attempt to control thoughts	4 (13.3)
Deterrents - *the young person identified things that*:	
Definitely stopped them from attempting suicide	5 (16.7)
Probably stopped them from attempting suicide	10 (33.3)
Most likely *did not* stop them from attempting suicide	4 (13.3)
Definitely *did not* stop them from attempting suicide	0 (0)
Uncertain	6 (20.0)
Reasons - *the young person wished to die*:	
Completely to get attention, revenge or a reaction from others	3 (10.0)
Mostly to get attention, revenge or a reaction from others	4 (13.3)
Equally to get attention, revenge or a reaction from others *and* to end pain	6 (20.0)
Mostly to end pain	9 (30.0)
Completely to end pain	8 (26.7)

Values are reported as *n* (%). *n* = sample size; C-SSRS, Columbia-Suicide Severity Rating Scale.

### 3.2 Sleep at baseline

On the CSHQ, most of the Autistic youth met the cutoff (a total score on the CSHQ of > 41) to be considered “poor sleepers”. Total sleep time was, on average, 8 h 46 min, which is within the recommended amount of time. However, based on the National Sleep Foundation’s (NSF) guidelines, 12 Autistic youth (21%) slept less than the recommended amount for their age, 10 youth slept less than 9 hours, and 2 youth slept less than 8 hours (32). Additionally, 3 youth (5%) slept more than the recommended amount, 2 youth slept more than 11 hours, and 1 youth slept more than 9 hours.

### 3.3 Sleep during the daily diaries

Forty-five (88.2%) of the recruited Autistic youth completed all seven days of the daily sleep survey, with an additional 5 (9.8%) completing six days and 1 participant completing only three days. All caregiver-reported sleep characteristics are presented in **Table 3**. Little’s Missing Completely at Random (MCAR) test (39) was not significant (*χ²* = 167.054, *p* = .176), indicating that the data were missing at random. Given the low proportion of missing data, analyses were conducted using participants with no missing data, and no imputation procedures were used.

**Table 3:** Caregiver-reported sleep characteristics (n=51).

Variable	Mean (SD)	*n* (%)
CSHQ ^a^ total score (n = 51)		
CSHQ Total Score Good sleepers (CSHQ ¾ 41) Poor sleepers (CSHQ > 41)	53.2 (10.68)	6 (12%) 44 (88%)
Sleep Diary (n = 51)		
Total Sleep Time (TST) Bedtime Wake time	8 h 46 min (2 h 33 min) 9:54 PM (1:53) 7:21 AM (1:14)	

Values are reported as Mean (SD) or *n* (%). *n*, sample size; SD, standard deviation; CSHQ, Children’s Sleep Habits Questionnaire; TST, total sleep time.

### 3.4 Sleep and lifetime suicidality

As outlined in **Table 4**, only two sleep variables were associated with lifetime suicide: daytime sleepiness and sleep quality. The daytime sleepiness subscale on the CSHQ was a significant predictor of group membership of the suicidal ideation variable, with those Autistic youth who experienced daytime sleepiness more likely to have experienced suicidal thoughts at some point in their lifetime. Specifically, increases in daytime sleepiness on the CSHQ were associated with an increase in the likelihood of reported suicidal ideation, with an odds ratio of 1.204 (95% CI [1.032, 1.405]). On average, Autistic youth who were reported to experience suicidal ideation had a score of 15.56 compared to 12.22 for those who did not. Sleep quality over the seven days was also related to suicidality, such that decreases in caregiver-reported sleep quality was associated with an increase in the likelihood of reported suicidal thoughts, with an odds ratio of 0.303 (95% CI (0.096, 0.953)), indicating that every decrease of 1 on the sleep quality rating increased the odds of suicidal ideation by approximately 3 times. On average, participants who reported suicidal ideation (*n* = 33) had a mean sleep quality of 2.08/3, while those who did not (*n* = 25) had a mean sleep quality of 2.439/3.

**Table 4:** Results of logistic regression models examining associations between sleep variables and suicidality outcomes.

Predictor	Suicidal ideation					Self-injury					Non-suicidal self-harm				
	*B*	*SE*	*OR*	*95% CI*	*p*	*B*	*SE*	*OR*	*95% CI*	*p*	*B*	*SE*	OR	*95% CI*	*p*
Daily Surveys															
Sleep Length	-0.12	0.21	0.89	0.59-1.34	.570	0.16	0.32	1.17	0.63-2.20	.600	.001	0.19	1	0.69-1.45	.990
Sleep Length Variability	0.37	0.38	1.45	0.69-3.05	.334	-0.62	0.66	0.54	0.15-1.96	.344	- 0.16	0.34	0.85	0.44-1.66	.640
Sleep Onset	-3.77	2.35	0.02	0.00-2.31	.114	-1.86	1.94	0.16	0.00-6.98	.341	-0.01	1.28	0.99	0.08-12.17	.990
Sleep Onset Variability	0.97	1.82	2.64	0.07-93.43	.531	-0.67	2.87	0.51	0.00-141.9	.810	-.46	1.68	0.63	0.02-16.90	.780
Sleep Quality	**-1.19**	**0.58**	**0.30**	**0.10-0.95**	**.040**	-0.23	0.79	0.79	0.17-3.74	.760	-0.11	0.48	0.9	0.35-2.30	.810
Sleep Quality Variability	0.31	0.93	1.36	0.22-8.44	.734	-3.72	2.03	0.02	0.00-1.30	.062	-0.61	0.90	0.54	0.09-3.17	.480
Baseline Surveys															
CSHQ Total Sleep Problems	0.04	0.03	1.04	0.98-1.10	.135	0.01	0.04	1.01	0.93-1.09	.924	-0.02	0.02	0.98	0.94-1.02	.450
Bedtime Resistance	0.04	0.09	1.04	0.87-1.24	.601	0.24	0.17	1.27	0.91-1.77	.141	0.05	.08	1.05	0.90-1.23	.540
Sleep Onset Delay	0.54	0.39	1.72	0.80-3.69	.151	-1.03	0.84	0.36	0.07-1.85	.220	0.09	0.36	1.09	0.54-2.22	.870
Sleep Duration	0.33	0.17	1.39	1.00-1.94	.052	0.08	0.25	1.08	0.66-1.77	.733	0.06	0.14	1.06	0.81-1.40	.670
Sleep Anxiety	0.07	0.11	1.07	0.86-1.33	.952	0.26	0.22	1.3	0.84-2.00	.224	-0.05	0.11	0.95	0.77-1.18	.620
Night Wakings	0.10	0.18	1.11	0.78-1.57	.552	0.23	0.27	1.26	0.74-2.14	.385	-0.29	0.18	0.75	0.53-1.06	.110
Parasomnias	0.02	0.13	1.02	0.79-1.32	.822	.001	0.27	1	0.59-1.70	1.00	0.01	0.12	1.01	0.80-1.28	.900
Sleep Breathing	-0.45	0.27	0.64	0.38-1.08	.102	0.39	0.75	1.48	0.34-6.42	.604	-0.28	0.29	0.76	0.43-1.33	.280
Daytime Sleepiness	**0.19**	**0.08**	**1.21**	**1.03-1.41**	**.011**	0.08	0.11	1.08	0.87-1.34	.464	0.09	0.06	1.09	0.97-1.23	.150

B, logistic regression coefficient; SE, standard error; OR, odds ratio; CI, confidence interval; CSHQ, Children’s Sleep Habits Questionnaire. Significant associations are indicated in bold (p <.05).

### 3.5 Sleep and recent suicidality

As shown in **Table 5**, the bedtime resistance and night wakings subscales of the CSHQ were associated with recent self-injury. More specifically, higher scores on the bedtime resistance subscale of the CSHQ, indicating more resistance, were associated with increased likelihood for caregivers to report self-injurious behaviours in their Autistic youth, with an odds ratio of 1.395 (95% CI (1.059, 1.838)). On average, Autistic youth who reported self-injury (*n* = 5) had a bedtime resistance score of 12.8 compared to 8.235 for those who did not (*n* = 34). Higher scores on the night wakings subscale of the CSHQ were associated with increased likelihood of caregiver-reported self-injurious behaviours, with an odds ratio of 2.081 (95% CI (1.005, 4.307)). On average, caregivers who reported self-injury in their Autistic youth had more night wakings with a score of 6.8 compared to 4.939 for those caregivers who did not report self-injury. The other sleep variables were not significantly related to suicidality.

**Table 5:** Results of logistic regression models examining associations between sleep variables and recent suicidality outcomes.

Predictor	Self-injury					Preparation behaviours				
	*B*	*SE*	*OR*	*95% CI*	*p*	*B*	*SE*	*OR*	*95% CI*	*p*
Daily Surveys										
Sleep Length	-0.40	0.29	0.67	0.38-1.18	.174	-0.40	0.26	0.67	0.40-1.12	.490
Sleep Length Variability	-0.05	0.59	0.95	0.30-3.02	.921	0.83	0.45	2.29	0.95-5.54	.060
Sleep Onset	2.62	3.24	13.74	0.02-7866	.422	-1.46	1.56	0.23	0.01-4.94	.350
Sleep Onset Variability	1.19	2.62	3.29	0.02-558	.653	2.64	2.19	14.01	0.19-1024	.230
Sleep Quality	-2.03	1.04	0.13	0.02-1.01	.051	**-3.4**	**1.21**	**0.03**	**0.00-0.36**	**.005**
Sleep Quality Variability	-0.55	1.67	0.58	0.02-15.23	.741	-0.44	1.28	0.64	0.05-7.92	.730
Baseline Surveys										
CSHQ Total	0.02	0.04	1.02	0.94-1.10	.542	**0.13**	**0.05**	**1.14**	**1.03-1.26**	**.008**
Bedtime Resistance	**0.33**	**0.14**	**1.39**	**1.06-1.83**	**.010**	0.12	0.11	1.13	0.91-1.40	.280
Sleep Onset Delay	0.34	0.66	1.4	0.39-5.12	.604	1.07	0.65	2.92	0.82-10.42	.100
Sleep Duration	0.58	0.34	1.79	0.92-3.48	.084	**0.54**	**0.26**	**1.72**	**1.03-2.86**	**.040**
Sleep Anxiety	0.34	0.19	1.4	0.97-2.04	.074	0.10	0.15	1.11	0.82-1.48	.510
Night Wakings	**0.73**	**0.37**	**2.08**	**1.00-4.29**	**.043**	0.40	0.26	1.49	0.90-2.48	.120
Parasomnias	0.23	0.18	1.26	0.88-1.79	.203	0.15	0.17	1.16	0.83-1.62	.350
Sleep Breathing	0.28	0.36	1.32	0.65-2.68	.431	0.14	0.34	1.15	0.59-2.24	.680
Daytime Sleepiness	0.03	0.10	1.03	0.85-1.25	.731	**0.23**	**0.10**	**1.26**	**1.03-1.53**	**.030**

B, logistic regression coefficient; SE, standard error; OR, odds ratio; CI, confidence interval; CSHQ, Children’s Sleep Habits Questionnaire. Significant associations are shown in bold (p <.05).

Additionally, the CSHQ total score, as well as mean sleep quality across the 7 daily surveys, were associated with recent preparation to attempt suicide. Increased CSHQ total scores were associated with increased likelihood to report preparation behaviours with an odds ratio of 1.140 (95% CI (1.035, 1.255)). This association was seemingly driven largely by the sleep duration and daytime sleepiness subscales. On average, Autistic youth whose caregivers reported preparation behaviours had a CSHQ score of 65.286 compared to 51.233 for those who did not. Decreased sleep quality across the 7 days was associated with increased likelihood of reporting recent preparation to attempt suicide with an odds ratio of 0.033 (95% CI (0.003, 0.356)), indicating that a 1-point decrease in sleep quality rating increased the odds of preparatory behaviours by approximately 30 times. On average, participants who reported preparation behaviours (*n* = 14) had a mean sleep quality rating of 1.476/3 compared to 2.323/3 for those who did not (*n* = 44). Within-subject variability in sleep across the 7 days was not significantly related to any aspect of suicidality.

### 3.6 Sleep and suicidality severity

The associations between the sleep variables and total suicidality severity scores in Autistic youth whose caregivers reported suicidal ideation are reported in **Table 6**. Of the sleep variables, only the daytime sleepiness subscale of the CSHQ was associated significantly with suicidality severity both in terms of lifetime scores (*R^2^* = .29) and recent scores (*R^2^* = .217).

**Table 6:** Linear regression models examining associations between sleep variables and C-SSRS suicidality severity scores.

Predictor	Lifetime C-SSRS total score				Recent C-SSRS total score			
	*B*	*SE*	*t*	*p*	*B*	*SE*	*t*	*p*
Daily Surveys								
Sleep Length	0.05	0.83	0.06	.942	0.19	0.80	0.23	.811
Sleep Length Variability	0.83	1.60	0.51	.601	0.32	1.56	0.20	.832
Sleep Onset	-1.68	5.24	-0.32	.754	1.39	5.10	0.26	.794
Sleep Onset Variability	3.87	7.82	0.49	.624	3.65	7.61	0.48	.630
Sleep Quality	-2.83	2.24	-1.26	.211	-2.01	2.20	-0.91	.364
Sleep Quality Variability	1.41	4.96	0.28	.771	3.73	4.79	0.79	.444
Baseline Surveys								
CSHQ Total	0.15	0.10	1.46	.155	0.112	0.10	1.09	.282
Bedtime Resistance	0.34	0.36	0.94	.353	0.351	0.31	1.00	.320
Sleep Onset Delay	1.38	1.81	0.76	.463	1.51	1.70	0.85	.390
Sleep Duration	0.84	0.67	1.25	.215	0.67	0.65	1.04	.301
Sleep Anxiety	0.45	0.52	0.86	.394	0.38	0.50	0.77	.441
Night Wakings	-0.88	0.75	-1.18	.245	-1.11	0.70	-1.61	.115
Parasomnias	-0.04	0.53	-0.08	.933	0.06	0.51	0.05	.961
Sleep Breathing	0.28	1.31	0.21	.839	0.42	1.25	0.33	.741
Daytime Sleepiness	**0.83**	**0.23**	**3.49**	**.001**	**0.69**	**0.24**	**2.88**	**.007**

B, regression coefficient; SE, standard error; t, t statistic; C-SSRS, Columbia-Suicide Severity Rating Scale; CSHQ, Children’s Sleep Habits Questionnaire. Significant associations are shown in bold (p <.05).

## 4 Discussion

Suicide is exceptionally common in Autistic people, with suicidal thoughts and behaviours emerging as young as 7 years of age (3, 4). Despite this, little is known about factors that might increase suicide risk in Autistic youth, which is critical information to inform prevention strategies. Sleep quality and quantity have been recognised as a transdiagnostic factor of suicide in Autistic and non-Autistic adults; yet, only a few studies have examined the sleep-suicidality association in Autistic youth (4, 14, 40). Thus, the current study is the first to comprehensively examine the association between sleep problems and suicidality in Autistic youth using both caregiver-reported questionnaires and daily sleep diaries.

Our study found that over half of caregivers reported that their Autistic youth experienced some form of suicidal behaviour and had engaged in NSSI in the last three months. Additionally, the majority of Autistic youth in our sample were “poor sleepers” and at risk for paediatric sleep disorders; yet, only twelve Autistic youth (21%) slept less than the recommended amount for their age, based on National Sleep Foundation’s recommendations (32), which is considerably better than previous reports in Autistic samples (16, 20, 41).

The current study also found that greater daytime sleepiness was associated with both lifetime and recent suicidality in Autistic youth. This aligns with studies in non-autistic adolescents reporting that excessive daytime sleepiness increases suicide risk (22, 42). Although the association between daytime sleepiness and suicidality is not fully understood, it has been proposed that excessive sleepiness impairs prefrontal-dependent inhibitory control and emotion regulation, which may lessen the ability to suppress suicidal thoughts or cope with distress (30, 43, 44). Further, lack of sleep and excessive sleepiness are known to impair prefrontal cognitive functioning (22, 42), reducing individuals’ ability to regulate emotions, inhibit negative thoughts, and manage suicidal thoughts. In Autistic youth, who often experience vulnerabilities in executive functioning and emotion regulation (42, 45), these factors may exacerbate and increase suicidal ideation, behaviour and death by suicide. Similarly, shorter sleep duration was also found to be associated with suicidality in our sample. Normal sleep duration is necessary to maintain optimal functioning and overall mental health (22, 23). Additionally, daytime sleepiness is linked to increased negative mood and social withdrawal (14). Feelings of fatigue can exacerbate irritability, hopelessness, and social disconnection, factors strongly associated with suicidal ideation in both Autistic and non-autistic populations (4, 43, 46). Furthermore, sleep loss can lead to cognitive rigidity and perseveration (27) or even worsening rumination linked to suicide (47). Overall, greater sleep-related problems may tax an Autistic individual’s ability to cope with life stressors and mental health conditions.

The hypothesis that total sleep problems would be significantly associated with suicidality was only partially supported by the results. Specifically, while daytime sleepiness and poorer sleep quality were associated with suicidality, the overall total sleep problems score (CSHQ Total) did not predict suicidal thoughts or behaviours (see **Table 4**). One possible explanation is that a CSHQ Total scores combines a range of sleep disturbances, some of which may not directly impact suicide and suicidal risk. For example, while short sleep duration and daytime sleepiness are linked to heightened suicide risk due to their impact on cognitive control, mood regulation, and fatigue (22, 23), other domains like parasomnias or sleep-disordered breathing (SDB) may not directly contribute to suicide and suicidal risk. Thus, certain sleep problems might drive suicide risk more than a general composite of sleep disturbances. Another explanation for our mixed findings may be that our study utilised caregiver-reported measures. In some instances, youth may be better at reporting both sleep problems and suicidality than caregivers. This has been highlighted by Storch, such that discrepancies in caregiver and child reports exist because children may be better reporters of internalising symptoms than their caregivers (48). Additionally, our younger sample could explain the discrepancies, as existing literature suggests that older youth are more likely to engage in suicidality (3).

The hypothesis that youth with a recent history of suicidality would have shorter sleep duration, longer night wakings, worse sleep quality, and later sleep times, assessed through daily sleep diaries, was partially supported by the results. Group differences indicated that Autistic youth with suicidality had significantly lower sleep quality scores (i.e., more fatigued), consistent with existing literature in the general population (22, 46). Further, youth in our sample with a history of suicidality were significantly older than their counterparts, consistent with findings in a sample of Autistic children (31.6%; 10–16 years versus 10.2%; 1–9 years) (3). This is consistent with developmental trends, as suicide risk increases during later adolescence due to increased social demands and identity formation challenges (49). For Autistic youth, adolescence in particular may amplify social isolation, bullying experiences, and mental health difficulties, all of which could exacerbate sleep problems and increase suicidality (4, 8). These results also align with the Interpersonal Theory of Suicide (IPTS), which posits that perceived burdensomeness and thwarted belongingness contribute to suicidal desire (9). In this context, sleep problems, especially those that impair daytime functioning, may exacerbate these interpersonal difficulties by increasing social withdrawal, limiting participation in daily activities, and intensifying feelings of being a burden, thereby compounding suicide risk.

While our study is cross-sectional, the findings raise questions about potential bidirectional links between sleep and suicidality in Autistic youth. Poor sleep may exacerbate emotional dysregulation and negative thinking patterns, increasing suicide risk, while suicidal ideation may in turn disrupt sleep, creating a vicious cycle. For example, Littlewood et al. (47) proposed that sleep disturbances could lead to perceived social isolation and burdensomeness, key drivers of suicidal ideation, while simultaneously impairing emotion regulation and impulse control, increasing the risk of suicidal behaviour. These mechanisms warrant examination in longitudinal studies.

The finding that sleep quality, but not total sleep duration, was related to suicidality suggests that subjective sleep experiences may be particularly important. For Autistic youth, whose sensory sensitivities and sleep architecture differences may alter sleep perceptions (41), understanding how they experience sleep could inform tailored interventions. For example, cognitive-behavioural therapy for insomnia (CBT-I), which targets maladaptive sleep cognitions and behaviours, may improve subjective sleep quality and potentially reduce suicide risk (46, 47).

Taken as a whole, results from the current study indicate that sleep problems are a potential risk factor for suicidal thoughts and behaviours in Autistic youth. Both sleep problems and suicidality are highly prevalent among Autistic youth. In a recent systematic review, Littlewood et al. (47) investigated psychological mechanisms linking sleep and suicidality and proposed that the IPTS, which emphasises perceived burdensomeness and social disconnection, may explain this link. Specifically, they suggest that sleep disturbances may exacerbate feelings of loneliness and lack of social connection, which are known risk factors for suicide. While loneliness and less caring relationships are not specific to autism, research does suggest that Autistic individuals experience greater social isolation compared to the general population (1, 9, 38, 49–51). It has also been hypothesised that sleep problems, such as insomnia, excessive daytime sleepiness, and hypersomnia, may increase the risk of suicide by impairing individuals’ ability to regulate emotions and increase the likelihood of impulsive behaviour (38, 52). Autistic individuals may already exhibit higher levels of impulsivity and difficulty regulating emotions, which may also predispose Autistic individuals to a heightened risk of suicide (45, 53). Prospective research is needed to further examine and clarify the complex mechanisms between sleep and suicidal thoughts and behaviours.

### 4.1 Strengths and limitations

There are several strengths of the current study, the first being the consideration of specific aspects of suicidality, including suicide ideation, suicidal behaviours and severity of suicide. Prior research has often relied on single-item measures of suicidality, limiting understanding of severity and multidimensional risk profiles (35, 41, 54). Our use of a modified C-SSRS allowed for a more nuanced assessment, although this measure has not been validated in Autistic populations (35). Furthermore, our study integrated a multi-method approach to assessing sleep, including cross-sectional measures and a seven-day sleep diary. Although objective measures are more reliable and consistent, using a multi-format approach allows for more reliable assessment of sleep than using a single tool (50).

The findings of the current study should be considered within the context of several limitations. As mentioned previously, the C-SSRS has not been designed for, nor validated in, Autistic populations. Because of the nature of the topic (i.e., suicide in children and adolescents) and the lack of available tools for Autistic youth (35), the questionnaire was modified for caregivers to answer questions about their children. Further, data was collected remotely with caregivers completing surveys at home, and we could not ensure real-time monitoring or immediate clinical response if youth disclosed acute suicidal thoughts. Therefore, we relied on caregiver reports to examine suicidality, limiting our data to thoughts and behaviours that could be detected by caregivers. Consequently, the current findings may have been an underrepresentation of the suicidality experiences of Autistic youth. Although this caregiver-report modification has not yet been validated in Autistic youth, evidence from related work suggests that caregiver-reported suicide measures can show reasonable agreement with youth-report scales. For instance, Flamarique et al. demonstrated that the caregiver-reported STOP-SAS showed fair accuracy in identifying suicidality (AUC = 0.756) and was moderately correlated with the original C-SSRS (r = 0.55, *p* <.001) in non-autistic populations (34). Furthermore, in their recent systematic review, Howe et al. (35) identified the C-SSRS as one of the most comprehensive tools for measuring suicide in the general population. Therefore, future research should attempt to gather both caregiver and youth-report assessments using validated tools (35). Although the sample was predominantly male, which is broadly consistent with Autistic trends, participants were also primarily White, and families who chose to participate may not fully represent the broader population of Autistic youth (4, 5). Therefore, the findings should be interpreted cautiously and may not generalize to more diverse Autistic populations or to youth experiencing more severe or acute suicidality.

Second, the current study used caregiver-reported measures of sleep instead of objectively measured sleep (i.e., actigraphy or polysomnography (PSG)). This may underestimate the true prevalence of sleep and suicidality. The use of the CSHQ in this study has further limitations. The CSHQ was initially developed for school-aged children (1, 3). Although parents of Autistic youth may monitor their children, including their sleep, there is limited empirical evidence quantifying how this affects reporting accuracy (18, 39, 51, 55). Future research should incorporate both subjective caregiver reports and objective sleep measures to improve accuracy in assessing sleep in Autistic populations.

Third, further limitations of the study include the small sample size (*n=57*) and cross-sectional design, which may have affected the results. For some analyses, only participants with a history of suicidality (*n=*35) were included, which lowers our power and makes the data susceptible to non-representativeness. Furthermore, bidirectional effects may exist; therefore, longitudinal studies are needed to clarify temporal pathways. Additionally, given the number of sleep variables examined across multiple regression models, there is an increased risk of Type I error due to multiple statistical tests, and the results should be interpreted with some caution pending further exploration and replication. Nonetheless, our results provide novel information regarding suicidality and sleep among Autistic children, youth, and young adults. Lastly, suicide risk and sleep experiences may differ for Autistic females and ethnic minorities, who face distinct social and structural stressors (11, 50). For example, Hedley and Uljarević identified lower socioeconomic status, lower parental education and being Black or Hispanic as risk factors for suicide in autism (50). Ethnic minorities and children from low SES families have different experiences, stressors, and resources than those from upper SES and non-minority households. Future research should aim to recruit larger and more diverse samples to better capture the range of experiences across socioeconomic, racial, and cultural groups.

### 4.2 Implications

This study has several clinical implications. Sleep problems in Autistic youth and young adults should be regularly screened for, especially during adolescence, a period of widespread shifts in the amount and type of sleep obtained (44, 56). Sleep problems may serve as observable and modifiable indicators of elevated suicide risk, and discussions about sleep may provide a less stigmatised entry point. Sleep problems at an early age may contribute to greater adverse outcomes due to biological and psychosocial changes during adolescence (44, 57, 58). By better understanding the relation between sleep problems and suicidality in Autistic youth, clinicians may be able to develop targeted intervention programs for high-risk children and adolescents. Assessment of sleep problems, including sleep duration and quality, should be screened for in Autistic youth, regardless of whether youth have endorsed suicidal thoughts. Additionally, severe sleep problems, such as daytime sleepiness and short sleep duration, should alert medical providers about the potential for an increased risk of suicide and other mental health concerns (59–61).

Second, interventions targeting sleep problems, such as CBT-I or sleep hygiene education, may have downstream benefits for mood, cognitive functioning, and suicidality (62). Given the transdiagnostic role of sleep in mental health, improving sleep could enhance emotion regulation and resilience to stress, thereby reducing suicide risk (57, 58).

Future research should employ longitudinal designs to clarify causal pathways between sleep problems and suicidality and incorporate multi-method assessments.

### 4.3 Conclusion

The present findings highlight a meaningful connection between sleep problems, especially daytime sleepiness and poor sleep quality, and suicidality in Autistic youth. Normative changes in sleep architecture during adolescence could help explain Autistic youths’ sleep experiences. Moving forward, it will be essential to determine whether strategies such as improving sleep hygiene, adapting cognitive-behavioural therapies for insomnia, creating predictable sleep environments, and supporting daily routines can enhance sleep quality and, in turn, reduce suicidal thoughts and behaviours. Although there appears to be a sleep-suicide association, additional research is needed to better clarify the mechanisms linking sleep problems to increased risk of suicidality in Autistic youth.

## Data Availability

Dataset includes information from caregivers and autistic youth and cannot be publicly shared. Requests to access the datasets should be directed to HS, hangsel.sanguino@ucalgary.ca.
